# Caudal and Lentiform nuclei Myelinolysis following Endoscopical surgery for pediatric Craniopharyngioma: two cases report and literature review

**DOI:** 10.1186/s41016-018-0125-8

**Published:** 2018-06-27

**Authors:** Zhi Gang Wang, Lian Ying Jiang, Wen Hao Zhang, Tao Hong

**Affiliations:** 1 Department of Neurosurgery, The First Affiliated Hospital of NanChang University, No.17 YongWai Street, NanChang, JiangXi 330006 People’s Republic of China; 2 Department of Neurology, Affiliated Hospital of Guangdong Medical University, Zhanjiang, Guangdong China; 3 Medical Image Center, Yongxin County People’s Hospital, JiAn, Jiangxi China

**Keywords:** Extrapontine myelinolysis(EPM), Craniopharyngioma, Endonasal endoscopic surgery, SIADH, Hypernatremia

## Abstract

**Background:**

The extrapontine myelinolysis (EPM) of osmotic demyelination syndrome (ODS) is a rare and dangerous disorder of the central nervous system. The mechanism is not fully understood and the treatment is controversial. There are few reports of EPM occurred after endonasal endoscopic resection of pediatric craniopharyngioma.

**Case presentation:**

We report two unusual cases of such a rare condition. Both are procedured uneventful endonasal endoscopical craniopharyngioma resection, after operation, the patients suffered serious diabetic insipidus with remarkable fluctuation of serum sodium though we applied pitutrin continual management to maintain everyday normal urine and correct serum sodium disorder. MR images occured caudal and lentiform nuclei myelinolysis following these serious conditions.

**Conclusions:**

In this study relevant literatures are reviewed in order to further understand this complication and contribute to the prevention and treatment of EPM caused by inappropriate correction of serum sodium disorder.

## Background

Craniopharyngioma is relatively rare benign intracranial tumor in children, with greater risks for surgery, because of its critic anatomic relation to hypothalamus. There are variety of complications after operation. Among these, diabetic insipidus (DI) and electrolyte disturbance are the most common complications. And osmotic demyelination syndrome have close relationship with rapid correction of serum sodium disorder after operation. It can cause serious symptoms [1], for example myelinolysis, seizure, disturbance of consiciousness, paralysis, and dyskinesia. It may be affect the prognosis of patients. Osmotic demyelination may occur in central pontine or extrapontine. For extrapontine demyelination, the basal ganglia region are often involved, it is particularly affected caudal and lentiform nuclei [2]. Above all, craniopharyngioma after surgery often cause electrolyte disturbance and diabetis insipidus, but the cases of osmotic demyelination followed by rapid correction of the electrolyte disturbance [2, 3], was so rare in previous reports. Today we report two cases of children who presented serious serum sodium disorder after endscopic surgery of craniopharyngioma. Both occured extrapontine myelinolysis followed by rapid correction of hyponatremia, but wether hyponatremia or hypernatremia can cause EPM still remained controversial. In this study we reviewed the relevant literatures and case reports to further discuss and understand the clinical feature, treatment and prevention of this syndrome.

## Case presentation

### Case 1

**Fig. 1:**
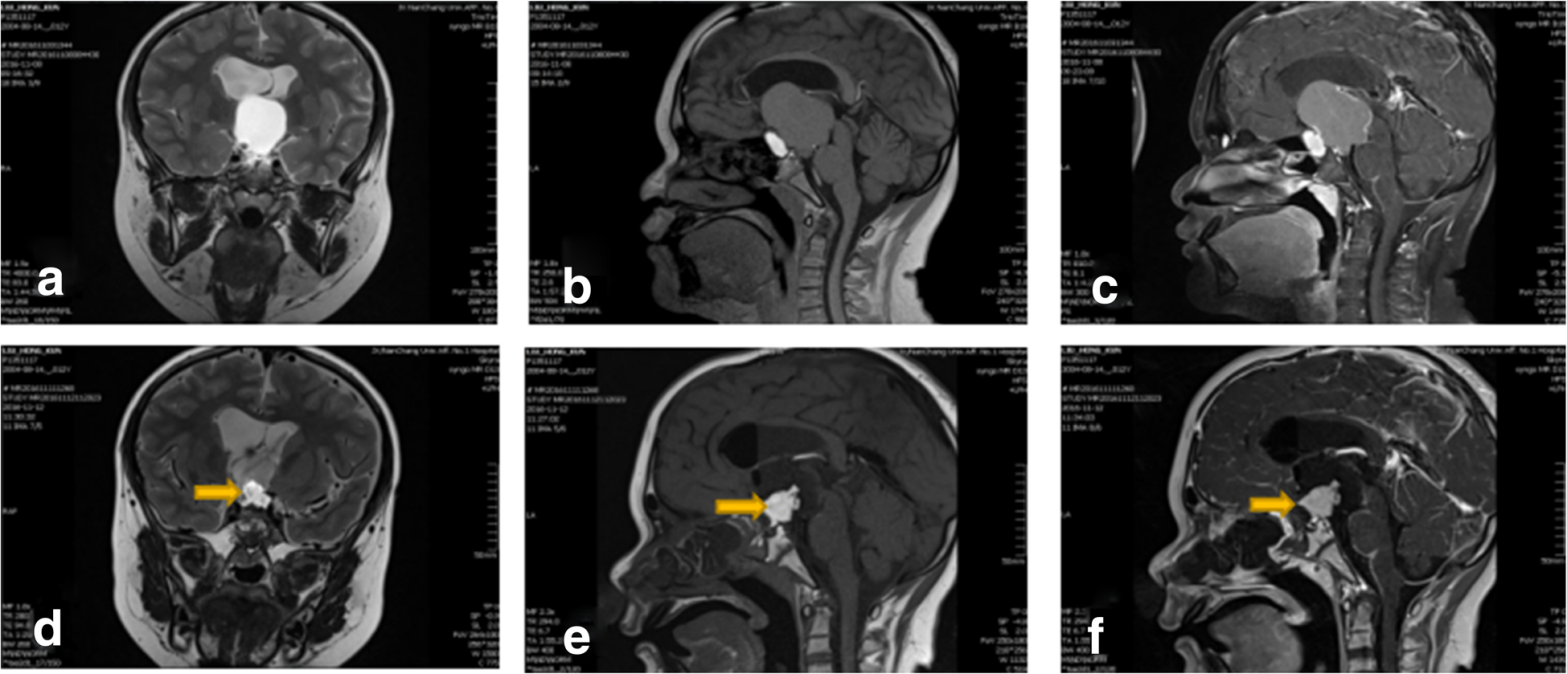
The pre- and post - operative MR images. **a**, **b**, **c**: coronal T2-weighted magnetic resonance image (**a**) and sagittal T1-weighted MR image (**b**) and sagittal T1-weighted MR with contrast image (**c**) taken before operation showed a huge cyst-solid craniopharyngioma lesion; **d**, **e**, **f**: coronal T2-weighted MR image (**d**) and sagittal T1-weighted MR image (**e**) and sagittal T1-weighted MR with contrast image (**f**) taken on postoperative day 1 showed that the tumor were total resection. The Yellow arrows showed auto-adipose tissue for repairing diaphragma sellae after tumor section

**Fig. 2:**
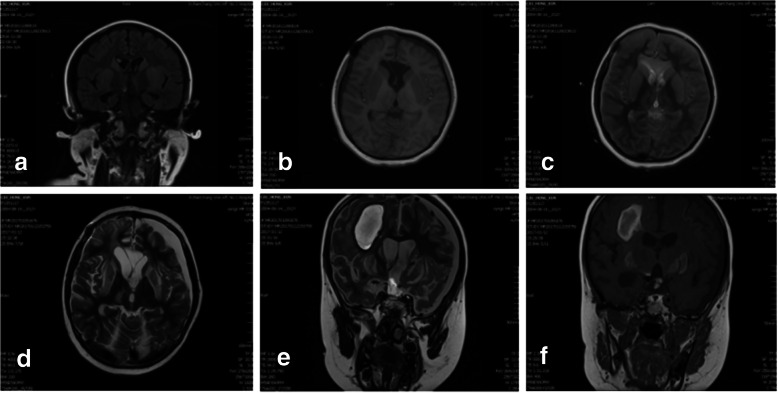
Postoperative day 16 and 30 MR images. **a**, **b**, **c**: The three images showed slightly symmetrical abnormal lesions at basal ganglia nucleus taken on postoperative day 16. Coronal FLAIR image (**a**) and axial T1-weighted MR image (**b**) show hypointense abnormality in the basal ganglia area. Axial T2-weighted MR image (**c**) showed slightly hyperintense change in the same areas. **d**, **e**, **f**: the three images were all taken on postoperative day 30. axial T2-weighted MR image (**d**) showed markedly symmetrical hyperintense abnormalities in caudate nucleus and lenticular nucleus; coronal T2-weighted (**e**) and T1-weighted (**f**) images showed a lesion in right frontal lobe with central hypointense and peripheral hyperintense signal, that was demyelination pseudotumor

The child patient, weighted 41Kg, a 12-year-old boy was admitted to hospital with headache for 1 year. He had undergone microscopic craniopharyngioma resection 7 years ago. The recent brain MR imaging showed tumor recurrence and obstructive hydrocephalus. The Fig. 1 shows the pre- and post- operative MR images. After a complete of medical evaluation, he accepted uneventful endonasal endoscopic surgery under general anesthesia in our department on Nov 11, 2016, and achieved total resection. After operation he kept a clear state of consciousness, but had persistent diabetes insipidus(DI). on the second day of postoperative, we applied pituitrin by continuous intravenous injection for 1 U per hour, in order to control 24 h urine volume between 2000 and 3000 ml. The serum sodium concentration began to fluctuate after operation. On postoperative day 2 laboratory values showed hypernatremia (163 mEq/l), and DI had worsened, so we continous to use pituitrin. But the leve of serum sodium droped to 112 mEq/l on postoperative day 7. It suggested that he developed syndrome of inappropriate secretion of antidiuretic hormone (SIADH). His 24 h fluid intake was restricted at 2000 ml - 2500 ml, balanced the volume of output. At the same time hypertonic saline was applied for refractory hyponatremia. We also stoped pituitrin continous infusion, but it failed because of DI. His serum sodium remained low (ranged, 112 - 114 mEq/l) for the next days. On postoperative day 9 his conscious leve deteriorated rapidly, associated with epileptic seizure and right upper limb involuntary movement. The laboratory values of serum sodium ranged from 112 to 172 mEq/l within 24 h. The Fig. 5 shows postoperative fluctuation of serum sodium leves, urine volume and clinical course. The hypernatramia continoued for 3 days, and then declined to normal leve after treatment. On postoperative day 16, his brain MR imaging showed symmetrical abnormalities in the extrapontine (Fig. 2). The patient’s coma state persisted for 2 months. Three months later, the consciousness improved, he can respond to verbal stimuli with eye opening. The postoperative day 16 and 30 MR images (Fig. 2).

### Case 2

**Fig. 3:**
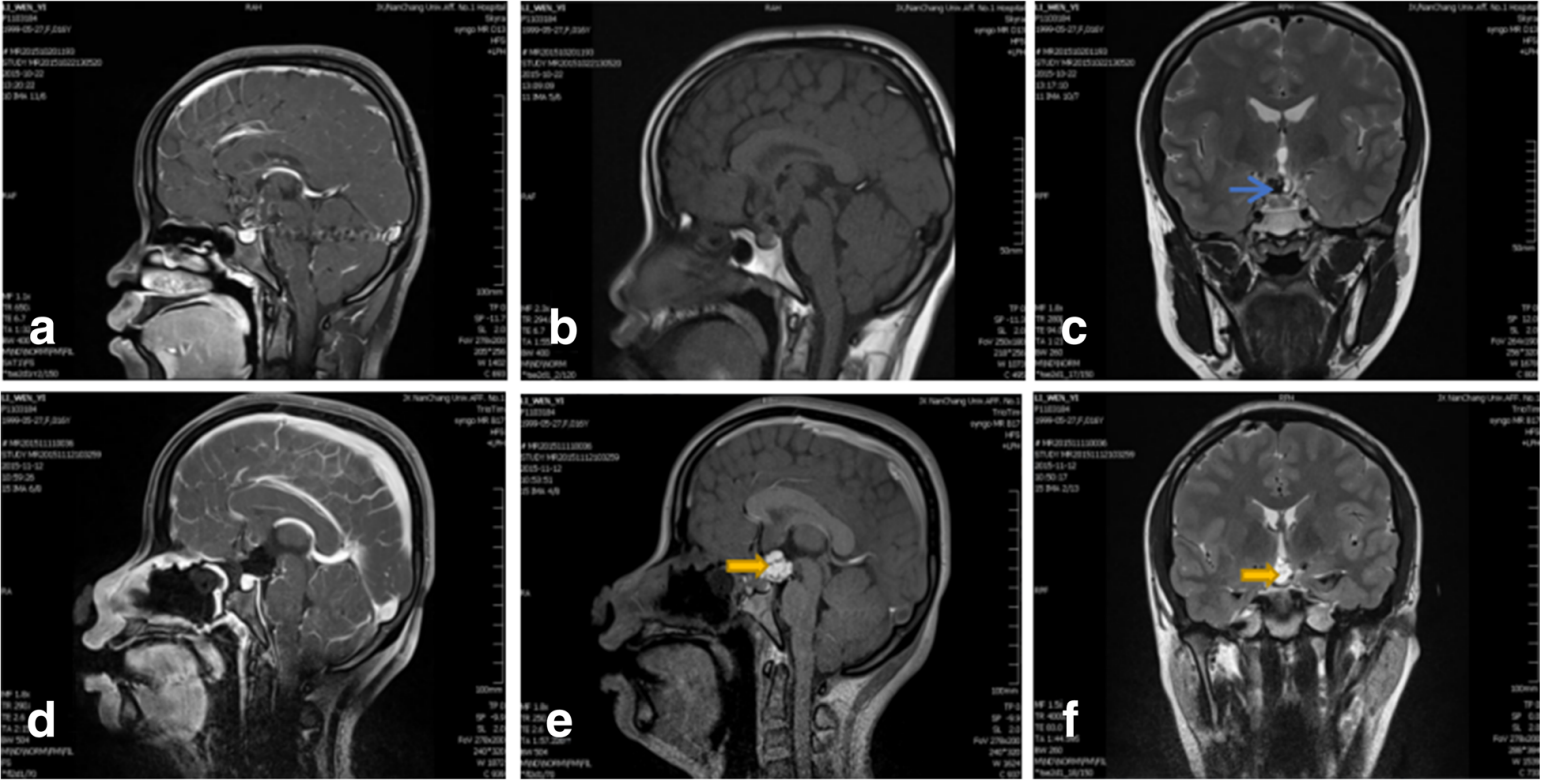
The pre- and post - operative MR images. **a**, **b**, **c**,: sagittal T1-weighted image with contrast enhancement and T1-weighted image (**a**, **b**) and coronal T2-weighted image (**c**) taken before endoscopic surgery showed a lesion in sellar area with calcification. **d**, **e**, **f**: the four images taken on postoperative day 2 showed the craniopharyngioma lesion was total resected. The green arrow showed calcification in tumor; the Yellow arrows showed auto-adipose tissue

**Fig. 4:**
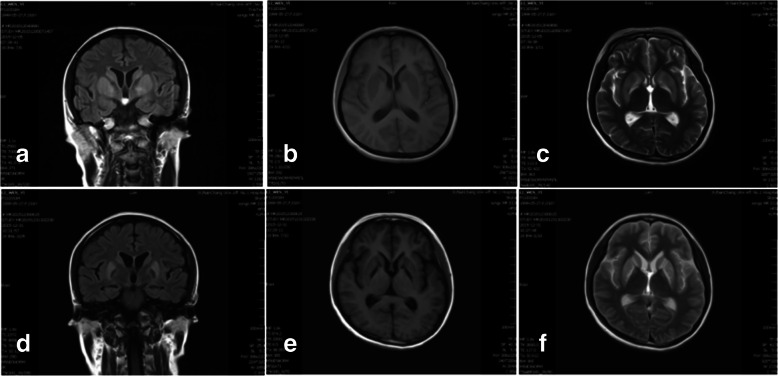
Postoperative day 20 and 49 MR images. **a**, **b**, **c**: coronal FLAIR MR image (**a**) taken on postoperative day 20 showed symmetrical bilateral hyperintense lesions in the lenticular nucleus. Axial T2-weighted MR image (**c**) showed markedly hyperintense signal in the same area. **d**, **e**, **f**: coronal FLAIR MR image (**d**) taked on postoperative day 49 showed the lesions with increased intensity and axial T1-weighted MR image showed hypointense abnormality in the basal ganglia area. Axial T2-weighted MR images (**f**) showed the lesions even worse, hyperintense lesions developed in bilateral caudate nucleus and lenticular nucleus

A 16-year-old girl, weighted 46KG, who was hospitalized with dizziness and headache for two months. And her brain MR imaging showed a lesion at the suprasellar region, considered craniophrayngioma. Fig. 3 shows pre- and post - operative MR images. She received endonasal neuroendoscopic operation to remove the suprasellar lesion on Nov 10, 2015. During the procedure, the lesion was total resection. On postoperative day 1, she was recovered well. But blood biochemical examination showed the sodium level was 168 mEq/l, and 24 h urine volume was about 3835 ml. Considered hypernatramia, we gave her hypotonic saline and water, at the same time applied pituirin to control urine output per hour. After treatment serum sodium concentration went down to the normal range. On day 5, the girl suffered accidental epileptic seizure, and also presented with dysarthria, gait ataxia and abnormal involuntary movement. Then her conscious leve deteriorated. The serum sodium concentration was 118 mEq/l at that time. We try our best to correct then and it still fluctuated between 115 to 118 mEq/l on day 6. On postoperative day 7 the serum sodium concentration went to 139 mEq/l suddenly. At the following 2 days it still went up persistent to 175 mEq/l. She was still at confusion. Hypernatramia state last for 3 days, and then declined to normal leve gradully. The MRI examination showed developed symertrical hypersignal at the lenticular nucleus in T2WI and FLAR compatible with extrapontine myelinolysis on the postoperative day 20 (Fig. 4). After one month rehabilitation execise, the symptoms were markedly improved, but still left mild dysarthria, gait ataxia. One year later she resumed her activity of preoperative daily living and went back to school life. Postoperative fluctuation of serum sodium leves, urine volume and clinical course (Fig. 6).

## Discussion

To our knowledge, there were only three reported cases about ODS following craniopharyngioma surgery [4–6] (Table 1). The causes of demyelination syndrome remains unknown. ODS has been generally observed in patient with malnutrition, diabetes, alcoholism and so on [4]. Sometimes latrogenic problems may be the important predisposing cause. But the majority of investigators considerd rapid correction of hyponatremia (12 mEq/(L.24 h)) lead to ODS, then occured EPM or CPM [7]. The risk of demyelination in central pontine and extrapontine increased when over corrected hyponatremia among 24 h [7]. This study presents two cases of pediatric craniopharyngioma developing extrapontine myelinolysis after endoscopic craniopharyngioma surgery who may be ralated to over-correction of hyponatremia. The mechanism of ODS associated with serum sodium rapid fluctuation is not clear. Intially, hyponatremia can cause brain edema and intracranial hypertension, induce cerebral perfusion decreasing, result in brain tissue hypoxia finally [8]. The nerve cells are very sensitive to hypoxia [8], especially for basal nuclei and central potine nerve axons, such as caudal and lentiform nuclei. Secondly, the fluctuation of serum osmotic pressure can give rise to central nervous demyelination. The rapid elevation of serum osmolarity caused cerebrovascular endothelial cells shrinkage, then damaged the blood brain barrier [9]. The substance exchange through vascular wall increased and damaged nerve myelin sheath cell. At the same time, the elevation of serum sodium concentration associated with serum osmotic changes by a large fluctuation, may be break up the steady state of cell volume [10]. It then developed central nervous myelinolysis diseases. And hypertonic stress leads to cell death in variety of cell types, particulaly oligodendrocytes [10]. Associated with the coexsiting factors, such as malnutrition, alcoholism, and so on, contribute to this process.

**Table 1:** Summary of reported cases of ODS following craniopharyngioma surgery

Case	Authors	Year	Age and Gender	Surgical approach	Initial symptoms	MRI	Medication	Outcome
1	I Kawahara et al. [4]	2009	18/F	_	Seizure, quardrip-aresis	CPM	_	Completely recovered
2	S Tsutsumi et al. [5]	2008	3/F	Endoscopic cystostomy	Seizure and coma	CPM EPM	_	Vegetative state
3	NA Sai Kiran et al. [6]	2014	6/M	Left pterional caniotomy GTE	Opisthotonic posturing	EPM	Syndopa, trihexyphenid-yl, baclofen	recovered

Abbreviations: *F*: Female, *M*: Male, *CPM*: Central pontine myelinolysis, *EPM*: Extrapontine myelinolysis, −: Not mentioned/not available, *GTE*: Gross total excision

**Fig. 5:**
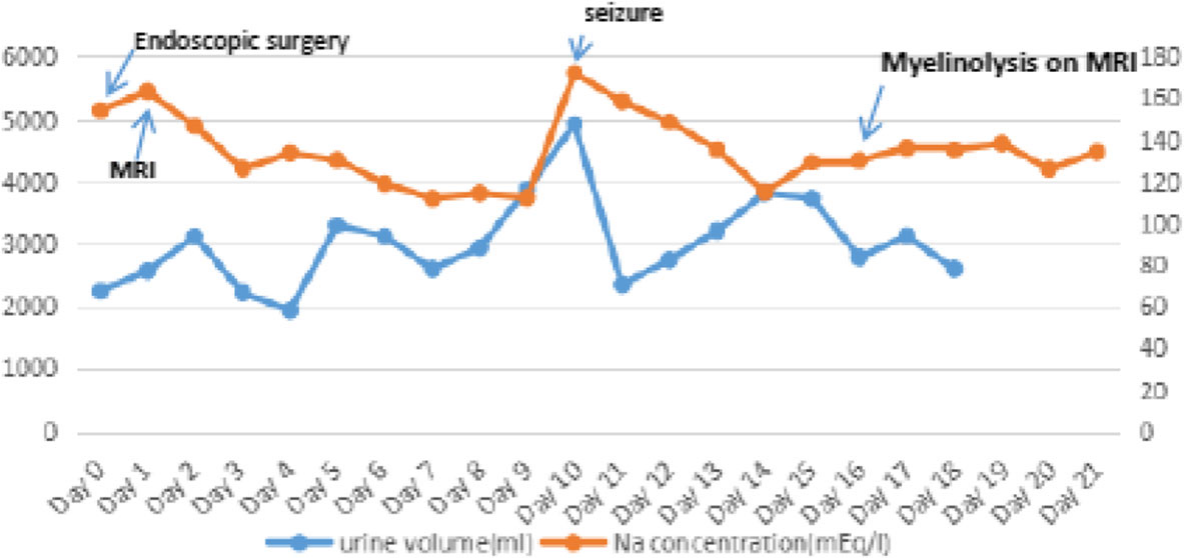
Postoperative fluctuation of serum sodium leves, urine volume and clinical course

**Fig. 6:**
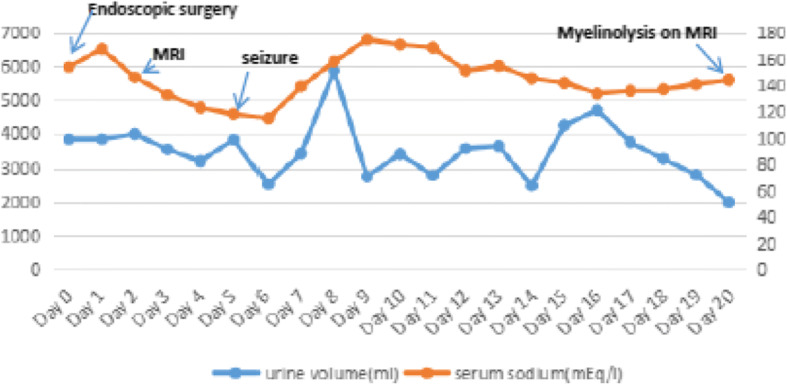
Postoperative fluctuation of serum sodium leves, urine volume and clinical course

The present 2 cases suffered craniopharyngioma, the surgical procedure itself was vulnerable to affect the vital structures, particularly hypothalamus and pituitary stalk. Although both children performed endoscopical surgery, it might have an exacerbating factor to vulnerable hypothalamus [5]. There were high occurrence rate of postoperative complications, especially for diabetes insipidus and electrolyte disturbance [5]. The child patient are vulnerable to disrupt the balance of whole body environment and increase the possibility of postoperative demyelinaton, because of the low volume of body fluid [5] and the large rate of body surface area. The two children in our study, both sufferd diabetes insipidus, and the variation trend of serum sodium presented three phases change (Figs. 5 and 6). On the early phase after operation, hypernatremia was main trends, it lasted for a few days, then followed by decrease to low level persistently. And the hyponatremia was very hard to correct, even if we applied pituitrin and the supplement sodium formula to do it. At this point, the amount of urine volume began to continuously decrease, but 24 h urine volume can still exceed or slightly lower than normal, associate with lower urine osmotic pressure, suggesting that abnormal secretion of antidiuretic hormone [3]. The changes of urine volume also consistent with serum sodium fluctuation (Figs. 5 and 6). Hypothalamic impairment likely to release over antidiuretic hormone on the phase of hyponatremia [11]. And at this time the urine volume had decreased, so we should restrict the fluid volume of input, in the condition of maintaining the balance of body fluid and monitored the concentration of serum sodium per day. It is important to confirm that the cause of hyponatremia [12], for each cause means different treatment strategy [12]. There at least exsits three main causes about hyponatremia, for example pesudohyponatremia, syndrome of inappropriate ADH(SIADH) and cerebral salt wasting syndrome(CSWS), as well as other uncommon reasons [12]. According to recent studies and recommendations suggestted that the velocity of correction was not in excess 8 mEq/(L.24 h) [3]. After the operation, all patients were clear awareness on the early stage, and there was a good correlation between the decrease of the state of consciousness and the rapid fluctuation of electrolyte, both suffered epileptic seizure and limb dyskinesia. But it might be controversial about the mechanism of ODS. Min Jee Hin et al. [13]. Reported ODS occured in acute hypernatremia, it leads to permanent neurologic symptoms. The craniopharyngioma patients suffered refractory hyponatremia after operation, which related to the syndrome of inappropriate ADH(SIADH) and cerebral salt wasting syndrome(CSWS) [11]. CSWS was the common cause of centrogenic hyponatremia, which might be overproduce brain natriuretic peptide after brain surgery or trauma. And brain natriuretic peptide might be cause renal tubular reabsorption of sodium ions decreased [12]. While SIADH is manly due to abnormal secretion of antidiuretic hormone increased kidney reabsorption of water, resulting in decreased body water discharge and blood volume increases [12]. For these two syndromes, we firstly should perform complete laboratory testing to verify accurate diagnosis. But fluid restriction is generally fist-line therapy in recent experts panel recommendations for SIADH [12], while CSWS is preferred to intravenous sodium supplementation [12]. Thomas E. Macmillan et al. Recent studies showed that patients treated with earlier administration of 1-Desamino-8d-Arginine-Vasopressin (DDAVP) were less likely to exceed the serum sodium correction limits recommended and might provide more reliable control of serum sodium [14]. But still it need more clinical datas to support it. At the same time, it is suggested that multidisciplinary management, such as pediatrics, department of endocrinology, neurology and so on, can help to reduce the incidence of postoperative complications.

To diagnosis of EPM, MR imaging examination may be the best choice because it is more sensitive than computed tomography(CT) [15]. In the acute stage, symmetric hyperintense signal on T2-weighted images and FLAIR are typical presentation (Figs. 2 and 4). Usually, the findings on the MRI can develop at least 2 to 3 weeks after appearance of initial symptoms [15], but in case1 first detected slightly hyperintense abnormalities within 1 week, and 2 weeks later it became so obvious on series of MR imaging (Fig. 2). The MR imaging presentation were consistent with other reports about ODS [4–6]. The differential diagnosis on MR imaging of EPM include brain edema, infarction, glioma, and other encephalopathy [15]. The severity of lesions on MRI was seemly correlated with the patients’ prognosis according to our cases and other literature showing on Table 1.

For demyelination diseases, at present, there are still no reliable methods of treatment. It need symptomatic treatment and intensive care for the patients, and also we suggest to use human gamma globulin, prednisone, nutrition and other strategy once the patient suffered the disease [6], supplemented by hyperbaric oxygen rehabilitation is also very effective. From our two cases, the case 1 the child have great recovered after discharged from our department 3 months later, he can respond to verbal with eye opening and crying. For the case 2, the child patient have returned to school and normal live since 3 months later after discharged from our department. EPM affected the prognosis of the patients, reduced the quality of life postoperation. Fewer child patients reported have positive recovery after active treatment, it suggested that ODS was potentially reversible in some consideration. But prevention is still the best strategy for EPM or ODS.

In summary, extrapontine myelinolysis is extremely rare conditions in pediatric after craniopharyngioma surgery, for the both child patients, rapid serum sodium fluctuation and DI on postoperative days was the main causes of dymelination syndrome. The management of serum sodium and DI after surgery is very important, larger fluctuation of serum sodium will increase the risk of dymelination syndrome. But it still need deeply research about this syndrome, for it always impact the children’s quality of life after surgery. Therefore, the possibility of developing ODS and EPM should be kept in mind when providing surgical treatment for such children.

## Conclusion

Extrapontine myelinolysis is extremely rare conditions in pediatric after craniopharyngioma surgery, for the both child patients, rapid serum sodium fluctuation and DI on postoperative days was the main causes of dymelination syndrome. The management of serum sodium and DI after surgery is very important, larger fluctuation of serum sodium will increase the risk of dymelination syndrome. But it still need deeply research about this syndrome, for it always impact the children’s quality of life after surgery. Therefore, the possibility of developing ODS and EPM should be kept in mind when providing surgical treatment for such children.

## Abbreviations

-: Not mentioned/not available
CPM: Central pontine myelinolysis
DI: Diabetic insipidus
EPM: Extrapontine myelinolysis
F: Female
GTE: Gross total excision
M: Male
MRI: Magnetic resonance imaging
ODS: Osmotic demyelination syndrome
SIADH: Syndrome of inappropriate secretion of antidiuretic hormone
